# Complete genome sequence of *Kangiella koreensis* type strain (SW-125^T^)

**DOI:** 10.4056/sigs.36635

**Published:** 2009-11-22

**Authors:** Cliff Han, Johannes Sikorski, Alla Lapidus, Matt Nolan, Tijana Glavina Del Rio, Hope Tice, Jan-Fang Cheng, Susan Lucas, Feng Chen, Alex Copeland, Natalia Ivanova, Konstantinos Mavromatis, Galina Ovchinnikova, Amrita Pati, David Bruce, Lynne Goodwin, Sam Pitluck, Amy Chen, Krishna Palaniappan, Miriam Land, Loren Hauser, Yun-Juan Chang, Cynthia D. Jeffries, Patrick Chain, Elizabeth Saunders, Thomas Brettin, Markus Göker, Brian J. Tindall, Jim Bristow, Jonathan A. Eisen, Victor Markowitz, Philip Hugenholtz, Nikos C. Kyrpides, Hans-Peter Klenk, John C. Detter

**Affiliations:** 1 DOE Joint Genome Institute, Walnut Creek, California, USA; 2 Los Alamos National Laboratory, Bioscience Division, Los Alamos, New Mexico, USA; 3 DSMZ - German Collection of Microorganisms and Cell Cultures GmbH, Braunschweig, Germany; 4 Biological Data Management and Technology Center, Lawrence Berkeley National Laboratory, Berkeley, California, USA; 5 Oak Ridge National Laboratory, Oak Ridge, Tennessee, USA; 6 Lawrence Livermore National Laboratory, Livermore, California, USA; 7 University of California Davis Genome Center, Davis, California, USA

**Keywords:** mesophile, non-pathogenic, aerobic and anaerobic growth, *Oceanospirillales*

## Abstract

*Kangiella koreensis* (Yoon *et al.* 2004) is the type species of the genus and is of phylogenetic interest because of the very isolated location of the genus *Kangiella* in the gammaproteobacterial order *Oceanospirillales. K. koreensis* SW-125^T^ is a Gram-negative, non-motile, non-spore-forming bacterium isolated from tidal flat sediments at Daepo Beach, Yellow Sea, Korea. Here we describe the features of this organism, together with the complete genome sequence, and annotation. This is the first completed genome sequence from the genus *Kangiella* and only the fourth genome from the order *Oceanospirillales*. This 2,852,073 bp long single replicon genome with its 2647 protein-coding and 48 RNA genes is part of the *** G****enomic* *** E****ncyclopedia of* *** B****acteria and* *** A****rchaea * project.

## Introduction

Strain SW-125^T^ (= DSM 16069 = KCTC 12182 = JCM 12317) is the type strain of the species *Kangiella koreensis,* which is the type species of the tiny (two species containing) genus *Kangiella* [1]. This genus was only recently identified (2004) in the course of screening microorganisms from a tidal flat of the Yellow Sea in Korea. The genus is named *Kangiella* in order to honor Professor Kook Hee Kang, a Korean microbiologist, for his contribution to microbial research. The species name pertains to Korea, from where the strain was isolated [1]. Although many moderately halophilic or halotolerant bacteria have been isolated and characterized taxonomically from this habitat [1], literature on *Kangiella* is very limited. Presently, the organism appears to be of interest solely for its position in the tree of life. Here we present a summary classification and a set of features for *K. koreensis* SW-125^T^ together with the description of the complete genomic sequencing and annotation.

### Classification and features

It is not evident from the taxonomic description of *K. koreensis* if any other strains beside SW-125^T^ have been isolated from this species. Uncultured clones with high 16S rRNA gene sequence similarity to the sequence of strain SW-125^T^ (AY520560) have been obtained from moderate saline crude oil contaminated soil in China (clone B109, 99%, EU328030). The highest degree of similarity to sequences from environmental metagenomic libraries [2] was only 91% (As of June 2009). 

**Figure 1:**
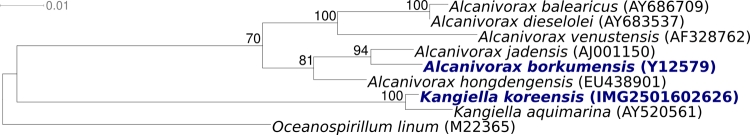
Phylogenetic tree highlighting the position of *K. koreensis* SW-125T relative to the other type strains in the phylogenetic neighborhood.. The tree was inferred from 1,476 aligned characters [3,4] of the 16S rRNA gene sequence under the maximum likelihood criterion [5], and rooted with the type strain of the order Oceanospirillales. The branches are scaled in terms of the expected number of substitutions per site. Numbers above branches are support values from 1,000 bootstrap replicates, if larger than 60%. Strains with a genome sequencing project registered in GOLD [6] are printed in blue; published genomes in bold.

Figure 1 shows the phylogenetic neighborhood of *K. koreensis* strain SW-125^T^ in a 16S rRNA based tree. Analysis of the two identical 16S rRNA gene sequences in the genome of strain SW-125^T^ differed by two nucleotides from the previously published 16S rRNA sequence generated from DSM 16069 (AY520560). The slight difference between the genome data and the reported 16S rRNA gene sequence is most likely due to sequencing errors in the previously reported sequence data.

**Figure 2:**
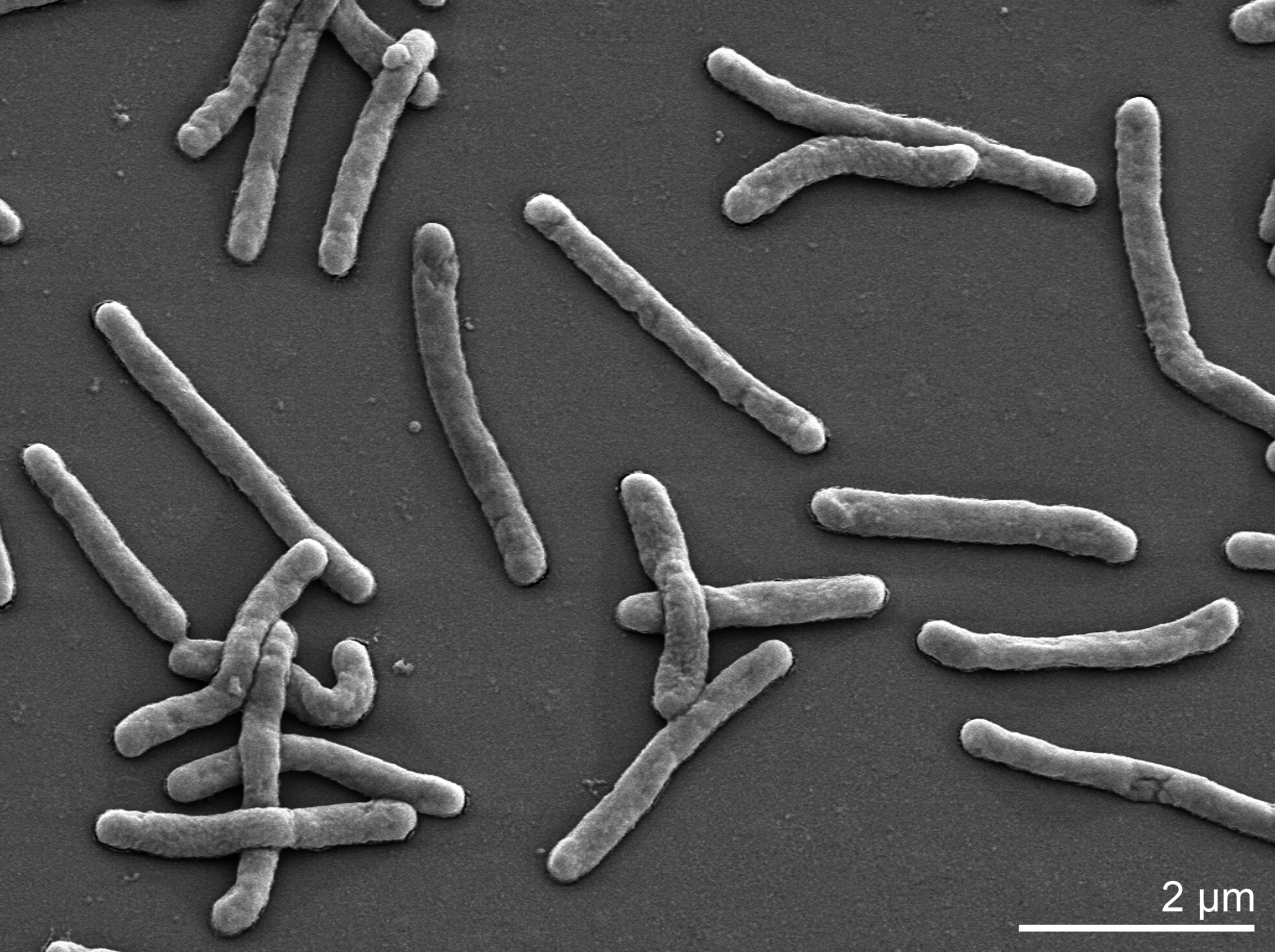
Scanning electron micrograph of *K. koreensis* SW-125^T^ (Manfred Rohde, Helmholz Centre for Infection Research, Braunschweig).

*K. koreensis* cells are rods of 0.2-0.5 × 1.5-4.5 µm in size (Table 1 and Figure 2). The colonies are smooth, raised, circular to irregular, light yellowish-brown in color and 2.0–3.0 mm in diameter after seven days incubation at 30°C on marine agar 2216 (MA) (Difco) [1]. The following physiological features are from Yoon *et al.* [1]. The growth conditions have been explored in quite detail. The growth at various temperatures was determined after incubation for at least 15 days on marine agar 2216 (Difco). The optimal growth temperature was at 30-37°C, with a minimum temperature of 4°C and a maximum temperature of 43°C [1]. The conditions of growth in dependence of pH were determined in marine broth 2216 (Difco). The optimal pH is 7.0 – 8.0. Growth is still possible at pH 5.5, but not at pH 5.0 [1]. Growth at various NaCl concentrations (1–15 %) was investigated in MB or trypticase soy broth (TSB, Difco). The optimal growth occurs in the presence of 2-3% NaCl (MB), growth still occurs in the presence of 12% NaCl (MB), but not without NaCl (TSB) or in the presence of more than 13% NaCl (MB) [1]. Growth under anaerobic conditions occurs on MA supplemented with nitrate. Strain SW-125^T^ hydrolyses casein, tyrosine, Tween 20, Tween 40 and Tween 60, but not Hypoxanthine and xanthine [1]. Furthermore, H_2_S is not produced, and nitrate is not reduced under aerobic conditions but to nitrogen gas under anaerobic conditions [1]. Acid is not produced from the following sugars: adonitol, L-arabinose, D-cellobiose, D-fructose, D-galactose, D-glucose, lactose, maltose, D-mannitol, D-mannose, D-melezitose, melibiose, *myo*-inositol, D-raffinose, L-rhamnose, D-ribose, D-sorbitol, sucrose, D-trehalose or D-xylose [1]. Unfortunately, a list of carbon sources from which acid is produced is not delivered [1]. When assayed with the API ZYM system, alkaline phosphatase, esterase (C4), esterase lipase (C8), leucine arylamidase, valine arylamidase, trypsin and naphthol-AS-BI-phosphohydrolase are present, but lipase (C14), cystine arylamidase, α-chymotrypsin, acid phosphatase, α-galactosidase, β-galactosidase, β-glucuronidase, α-glucosidase, β-glucosidase, N-acetyl-β-glucosaminidase, α-mannosidase and α-fucosidase are absent [1]. Strain SW-125^T^ was found to be susceptible to polymyxin (50 U), streptomycin (50 µg), penicillin (20 U), chloramphenicol (50 µg), ampicillin (10 µg), cephalothin (30 µg) and erythromycin (15 µg), and to be resistant to novobiocin (5 µg) and tetracycline (30 µg) [1].

**Table 1:** Classification and general features of *K. koreensis* SW-125^T^ according to the MIGS recommendations [7].

**MIGS ID**	**Property**	**Term**	**Evidence code**
	Current classification	Domain *Bacteria*	TAS [8]
		Phylum *Proteobacteria*	TAS [9]
		Class *Gammaproteobacteria*	TAS [10,11]
		Order *Oceanospirillales*	TAS [12,11
		Family *Incertae sedis*	NAS
		Genus *Kangiella*	TAS [1]
		Species *Kangiella koreensis*	TAS [1]
		Type strain SW-125	
	Gram stain	negative	TAS [1]
	Cell shape	rods, 0.2-0.5 × 1.5-4.5 µm	TAS [1]
	Motility	nonmotile	TAS [1]
	Sporulation	non-sporulating	TAS [1]
	Temperature range	4-43°C	TAS [1]
	Optimum temperature	30-37°C	TAS [1]
	Salinity	requires 2-3% (w/v) NaCl, growth at 12% but not 13% NaCl	TAS [1]
MIGS-22	Oxygen requirement	aerobic and anaerobic growth	TAS [1]
	Carbon source	no specific information available	
	Energy source	peptone	TAS [1]
MIGS-6	Habitat	tidal flats	TAS [1]
MIGS-15	Biotic relationship	free living	NAS
MIGS-14	Pathogenicity	unknown	
	Biosafety level	1	TAS [13]
	Isolation	tidal flat sediment	TAS [1]
MIGS-4	Geographic location	Daepo Beach, Yellow Sea, Korea	TAS [1]
MIGS-5	Sample collection time	2004 or before	TAS [1]
MIGS-4.1 MIGS-4.2	Latitude, Longitude	33.245, 126.409	NAS
MIGS-4.3	Depth	not reported	
MIGS-4.4	Altitude	not reported	

Evidence codes - IDA: Inferred from Direct Assay (first time in publication); TAS: Traceable Author Statement (i.e., a direct report exists in the literature); NAS: Non-traceable Author Statement (i.e., not directly observed for the living, isolated sample, but based on a generally accepted property for the species, or anecdotal evidence). These evidence codes are from the Gene Ontology project [14]. If the evidence code is IDA, then the property was observed for a living isolate by one of the authors or an expert mentioned in the acknowledgements.

Information on the composition of the peptidoglycan composition is unavailable. The predominant respiratory lipoquinone of *K. koreensis* SW-125^T^ is the ubiquinone Q-8 (comprising approximately 84-88%) [1]. The fatty acids comprise iso-C_11:0_ (5.6%), iso-C_13:0_ (0.4%), iso-C_15:1_ F (1.2%), iso-C_15:0_ (57.6%), iso-C_16:0_ (0.7%), C_16:0_ (1.1%), iso-C_17:0_ (7.2%), iso-C_17:1_ ω 9c (8.6%), iso-C_11:0_ 3-OH (10.5%), iso-C_17:1_ ω9c (8.6%), iso-C_11:0_ 3-OH(10.5%), iso-C_15:0_ 3-OH (0.9%), iso-C_17:0_ 3-OH (1.0%) and summed feature 1 (iso-C_15:1_ and/or C_13:0_ 3-OH) (3.2%) [1]. The predominance of iso-branched chain fatty acids indicates that the initial step in fatty acid synthesis is determined by an enzyme with a high degree of specificity for branched chain precursors (rather than acetate). The polar lipids of neither members of this species nor members of this genus have been investigated.

## Genome sequencing and annotation

### Genome project history

This organism was selected for sequencing on the basis of its phylogenetic position, and is part of the *** G****enomic* *** E****ncyclopedia of* *** B****acteria and* *** A****rchaea * project. The genome project is deposited in the Genomes OnLine Database [12] and the complete genome sequence in GenBank. Sequencing, finishing and annotation were performed by the DOE Joint Genome Institute (JGI). A summary of the project information is shown in Table 2.

**Table 2:** Genome sequencing project information

**MIGS ID**	**Property**	**Term**
MIGS-31	Finishing quality	Finished
MIGS-28	Libraries used	Two genomic libraries: one 8 kb pMCL200 Sanger library and one 454 pyrosequence standard library
MIGS-29	Sequencing platforms	ABI3730, 454 GS FLX
MIGS-31.2	Sequencing coverage	8.6x Sanger; 41× pyrosequence
MIGS-30	Assemblers	Newbler version 1.1.02.15, phrap
MIGS-32	Gene calling method	Prodigal
	INSDC ID	CP001707
	INSCD date of release	August 28, 2009
	GOLD ID	Gc01097
	INSDC project ID	29443
	Database: IMG-GEBA	2501533215
MIGS-13	Source material identifier	DSM 16069
	Project relevance	Tree of Life, GEBA

### Growth conditions and DNA isolation

*K. koreensis* SW-125^T^, DSM 16069, was grown in DSMZ medium 514 (BACTO Marine Broth) [15] at 28°C. DNA was isolated from 0.5-1 g of cell paste using Qiagen Genomic 500 DNA Kit (Qiagen, Hilden, Germany) following the manufacturer’s protocol, but with a modification ‘L’ for cell lysis, as described in Wu *et al.* [16].

### Genome sequencing and assembly

The genome was sequenced using a combination of Sanger and 454 sequencing platforms. All general aspects of library construction and sequencing performed at the JGI can be found at the JGI website (http://www.jgi.doe.gov/). 454 Pyrosequencing reads were assembled using the Newbler assembler version 1.1.02.15 (Roche). Large Newbler contigs were broken into 3,167 overlapping fragments of 1,000 bp and entered into the assembly as pseudo-reads. The sequences were assigned quality scores based on Newbler consensus q-scores with modifications to account for overlap redundancy and to adjust inflated q-scores. A hybrid 454/Sanger assembly was made using the parallel phrap assembler (High Performance Software, LLC). Possible mis-assemblies were corrected with Dupfinisher or transposon bombing of bridging clones [17]. Gaps between contigs were closed by editing in Consed, custom primer walk or PCR amplification. 329 Sanger finishing reads were produced to close gaps, to resolve repetitive regions, and to raise the quality of the finished sequence. The final assembly consists of 24,350 Sanger and 478,372 pyrosequence (454) reads. Together all sequence types provided 49.6x coverage of the genome. The error rate of the completed genome sequence is less than 1 in 100,000.

### Genome annotation

Genes were identified using Prodigal [18] as part of the Oak Ridge National Laboratory genome annotation pipeline, followed by a round of manual curation using the JGI GenePRIMP pipeline (http://geneprimp.jgi-psf.org/) [19]. The predicted CDSs were translated and used to search the National Center for Biotechnology Information (NCBI) nonredundant database, UniProt, TIGRFam, Pfam, PRIAM, KEGG, COG, and InterPro databases. Additional gene prediction analysis and functional annotation was performed within the Integrated Microbial Genomes Expert Review platform (http://img.jgi.doe.gov/er) [20].

## Genome properties

**Figure 3:**
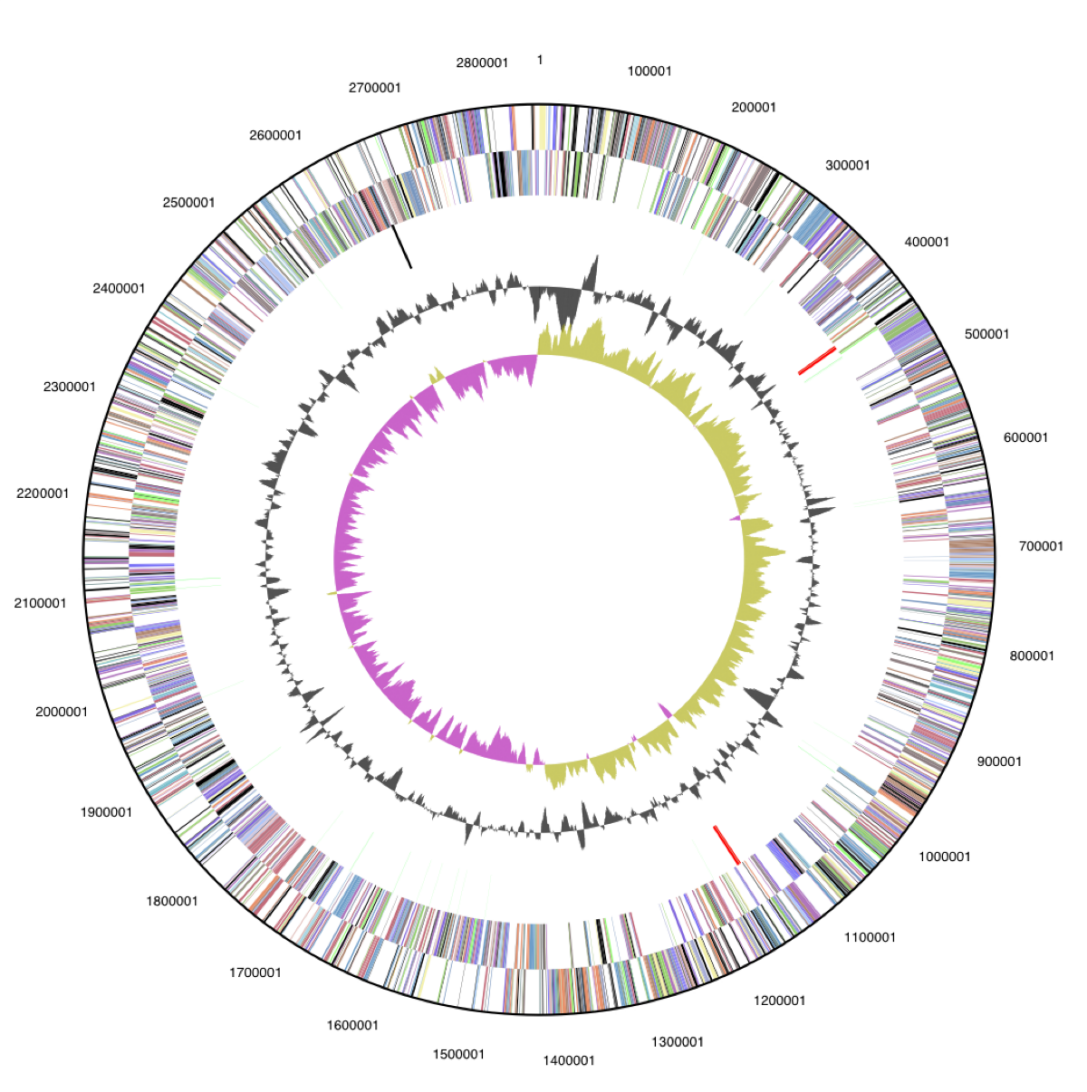
Graphical circular map of the genome. From outside to the center: Genes on forward strand (color by COG categories), Genes on reverse strand (color by COG categories), RNA genes (tRNAs green, rRNAs red, other RNAs black), GC content, GC skew.

The genome is 2,852,073 bp long and comprises one main circular chromosome with a 43.7% GC content. (Table 3 and Figure 3). Of the 2,695 genes predicted, 2,647 were protein coding genes, and 48 RNAs; 14 pseudogenes were also identified. The majority of the protein-coding genes (71.7%) were assigned a putative function while those remaining were annotated as hypothetical proteins. The distribution of genes into COGs functional categories is presented in Table 4.

**Table 3:** Genome Statistics

**Attribute**	Value	% of Total
Genome size (bp)	2,852,073	100.00%
DNA Coding region (bp)	2,585,246	90.64%
DNA G+C content (bp)	1,245,988	43.69%
Number of replicons	1	
Extrachromosomal elements	0	
Total genes	2,695	100.00%
RNA genes	48	1.78%
rRNA operons	2	
Protein-coding genes	2,647	98.22%
Pseudo genes	14	0.52%
Genes with function prediction	1,932	71.69%
Genes in paralog clusters	163	6.05%
Genes assigned to COGs	2,034	75.47%
Genes assigned Pfam domains	1,995	74.03%
Genes with signal peptides	691	25.64%
Genes with transmembrane helices	727	26.98%
CRISPR repeats	0	

**Table 4:** Number of genes associated with the general COG functional categories

**Code**	**Value**	**% age**	**Description**
J	170	6.4	Translation, ribosomal structure and biogenesis
A	1	0.1	RNA processing and modification
K	129	4.9	Transcription
L	106	4.0	Replication, recombination and repair
B	0	0.0	Chromatin structure and dynamics
D	29	1.1	Cell cycle control, mitosis and meiosis
Y	0	0.0	Nuclear structure
V	30	1.1	Defense mechanisms
T	134	5.1	Signal transduction mechanisms
M	139	5.3	Cell wall/membrane biogenesis
N	40	1.5	Cell motility
Z	1	0.0	Cytoskeleton
W	0	0.0	Extracellular structures
U	81	3.1	Intracellular trafficking and secretion
O	130	4.9	Posttranslational modification, protein turnover, chaperones
C	141	5.3	Energy production and conversion
G	41	1.5	Carbohydrate transport and metabolism
E	197	7.4	Amino acid transport and metabolism
F	54	2.0	Nucleotide transport and metabolism
H	118	4.5	Coenzyme transport and metabolism
I	84	3.2	Lipid transport and metabolism
P	113	4.3	Inorganic ion transport and metabolism
Q	53	5.3	Secondary metabolites biosynthesis, transport and catabolism
R	235	8.9	General function prediction only
S	223	8.4	Function unknown
-	613	23.2	Not in COGs
